# Fatal autoimmune pneumonitis requiring bilobectomy and omental flap repair in a patient with autoimmune polyendocrinopathy-candidiasis-ectodermal dystrophy (APECED)

**DOI:** 10.1016/j.rmcr.2021.101476

**Published:** 2021-07-08

**Authors:** Stephanie A. Kubala, Huy M. Do, Elise M.N. Ferré, David S. Schrump, Kenneth N. Olivier, Jeffrey G. Walls, Michail S. Lionakis, Les R. Folio

**Affiliations:** a National Institute of Allergy and Infectious Diseases, National Institutes of Health, Bethesda, MD, USA; b Radiology and Imaging Sciences, National Institutes of Health, Bethesda, MD, USA; c Center for Cancer Research, National Cancer Institute, Bethesda, MD, USA; d Pulmonary Branch, National Heart, Lung, and Blood Institute, National Institutes of Health, Bethesda, MD, USA; e Pulmonary and Critical Care, Novant Health, Charlotte, NC, USA

**Keywords:** APECED, APS-1, AIRE, Autoimmune pneumonitis, Bronchiectasis, Bronchopleural fistula, Omental flap repair, Immunomodulation

## Abstract

We present a severe case of progressive autoimmune pneumonitis requiring surgical intervention in a patient with the monogenic syndrome, autoimmune polyendocrinopathy-candidiasis-ectodermal dystrophy (APECED). APECED is caused by loss-of-function mutations in the autoimmune regulator (*AIRE*) gene, which lead to impaired central immune tolerance and autoimmune organ destruction including pneumonitis, an underrecognized, life-threatening complication. When clinicians evaluate patients with pneumonitis, recurrent mucosal candidiasis, and autoimmunity, APECED should be considered in the differential. Additionally, in patients with established APECED, a chest computed tomography is preferred to identify pneumonitis early on and to promptly initiate lymphocyte-directed immunomodulatory treatment, which can prevent irreversible lung destruction.

## Nomenclature

APECED: Autoimmune polyendocrinopathy-candidiasis-ectodermal dystrophy
CMC: Chronic mucocutaneous candidiasis
APS-1: Autoimmune polyglandular syndrome type 1
AIRE: Autoimmune regulator
TSAs: Tissue-specific antigens
mTECs: Medullary thymic epithelial cells
BPIFB1: Bactericidal/permeability-increasing fold-containing B1
GGO: Ground glass opacities
TIB: Tree-in-bud
NTM: Nontuberculous mycobacteria

## 1 Introduction

Autoimmune polyendocrinopathy-candidiasis-ectodermal dystrophy (APECED), also known as autoimmune polyglandular syndrome type 1 (APS-1), is a rare monogenic autoimmune disorder classically characterized by chronic mucocutaneous candidiasis (CMC) and multiorgan autoimmunity [1,2]. APECED results from biallelic mutations in the autoimmune regulator (*AIRE*) gene [[1], [2], [3], [4]]. *AIRE* plays a critical role in immune tolerance by directing expression of tissue-specific antigens (TSAs) in medullary thymic epithelial cells (mTECs) [1,[3], [4], [5]]. Naïve T-lymphocytes that recognize TSAs with high affinity undergo apoptosis, leading to negative selection and protection from autoimmunity. A dysfunction in this process leads to impaired central tolerance and escape of autoreactive T-lymphocytes into the periphery where they cause organ-specific autoimmune destruction [[1], [2], [3], [4], [5]]. Additionally, autoreactive B-lymphocytes have been shown to produce a variety of autoantibodies and to prime T-lymphocytes, worsening organ-specific damage [[6], [7], [8], [9], [10]].

While autoimmunity of endocrine organs is highlighted within the name of this disorder, non-endocrine manifestations are more common than originally thought [[11], [12], [13], [14]]. For example, initially described to occur in only a small subset (~2%) of APECED patients [15,16], pneumonitis was identified in 42% of patients in a prospective observational natural history study of 50 APECED patients [12,17]. Autoantibodies against the lung-targeted bactericidal/permeability-increasing fold-containing B1 (BPIFB1) and the potassium channel regulator KCNRG are highly specific for APECED pneumonitis development, although not all patients with biopsy-proven pneumonitis harbor these autoantibodies [12,[17], [18], [19], [20]]. APECED pneumonitis features a characteristic compartmentalized immunopathology with activated neutrophils in the airways, and T and B-lymphocyte infiltration within intraepithelial, submucosal, peribronchiolar, and interstitial areas of lung tissue [12,17].

Clinically, APECED pneumonitis presents with chronic respiratory symptoms, most often persistent cough with or without sputum production [12,17]. However, a proportion of patients (<5–10%) can be asymptomatic [12,17]. On imaging, APECED pneumonitis features ground glass opacities (GGO) and/or tree-in-bud (TIB) patterns and, if left untreated, it can progress to bronchiectasis and structural lung disease [12,17,18]. Progressive lung tissue destruction leads to significant morbidity and mortality. Early diagnosis is paramount to prevent irreversible structural organ damage [17]. Treatment of autoimmune pneumonitis with lymphocyte-directed combination of azathioprine (or mycophenolate mofetil) and rituximab has been shown to remit the disease [12]. Here, we present a case depicting the severe progression of APECED pneumonitis in a patient followed at our institution for over seven years.

## 2 Case presentation

**Fig. 1:**
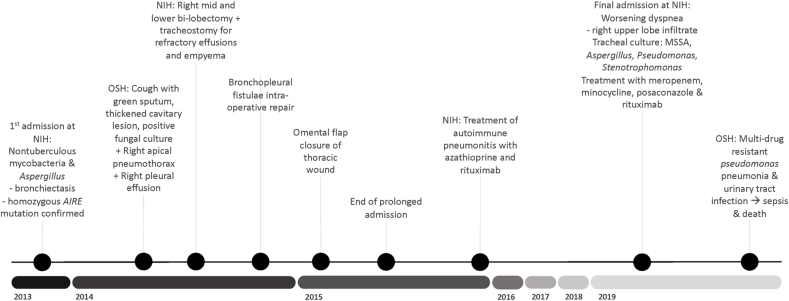
Clinical course timeline of the patient presented in our study. OSH: outside hospital.

A 50-year-old male with clinical APECED was referred to our institution for management of refractory pulmonary nontuberculosis mycobacterial (NTM) infection (Fig. 1). He was enrolled in the NIAID (11-I-0187) IRB-approved protocol and was provided written informed consent in accordance with the Declaration of Helsinki. His APECED manifestations included CMC diagnosed at age 3, Sjogren's-like syndrome (age 3), intestinal dysfunction (age 5), hypoparathyroidism (age 6), enamel hypoplasia (age 7), Addison's disease (age 14), pernicious anemia (age 20), vitiligo (age 35), and hypogonadism (age 52). He met clinical diagnostic criteria for APECED at 6 years of age upon development of CMC and hypoparathyroidism, and APECED was genetically confirmed upon NIH admission by Sanger sequencing of the *AIRE* gene, which identified homozygous c.967_979del13, p.L323SfsX51 (Fig. 1).

Our patient's respiratory problems began at 5 years of age as chronic daily, dry cough that progressed to recurrent episodes of bronchitis. At age 40 years, he developed daily cough productive of greenish purulent sputum and radiographic evidence of worsening bronchiectasis. At age 48 years, sputum culture grew *Mycobacterium avium* complex (MAC) with multiple cystic changes noted on chest CT. He was treated with rifampin, azithromycin, and ethambutol at an outside hospital. Despite triple antibiotic therapy, his symptoms persisted, sputum cultures remained positive for MAC, and chest CT identified coalescence of two areas of cystic bronchiectasis, which prompted the addition of amikacin. However, his clinical symptoms continued to worsen, leading to a referral to NIH.

**Fig. 2:**
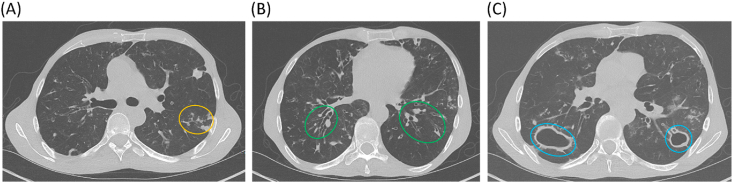
Imaging findings of autoimmune pneumonitis during first admission at the NIH. A. Radiographic features of APECED pneumonitis with ground glass opacities (GGO), tree-in-bud (TIB) opacities without bronchiectasis (yellow oval), and small nodular opacities. B. Bilateral bronchiectasis-associated structural lung disease and mucus plugging (green ovals). C. Large cavitary lesions caused by nontuberculous mycobacteria (NTM) (blue ovals). Chest CT performed in 2013. (For interpretation of the references to colour in this figure legend, the reader is referred to the Web version of this article.)

Non-contrast chest CT upon NIH admission showed a broad spectrum of findings representing early (GGO and tree-in-bud abnormalities), intermediate (bronchiectasis and nodules), and late stage characteristics (severe, diffuse bronchiectasis and cavity formation) of autoimmune lung disease complicated by infection (Fig. 2) [12,17]. Sputum culture grew *Aspergillus fumigatus*, methicillin-susceptible *Staphylococcus aureus* (MSSA) and MAC for which posaconazole was added for antifungal coverage, rifampin was discontinued due to drug interactions with posaconazole, and linezolid was initiated to optimize MAC therapy and treat MSSA. He was discharged home with close follow-up by local providers.

A year later, his clinical condition deteriorated, requiring daily home oxygen therapy with worsening cough and night sweats (Fig. 1). Evaluation at an outside institution was notable for right lower lobe cavity thickening and a plethora of microorganisms cultured from sputum including: *Candida albicans, Candida dubliniensis, Graphium* species, *Exophiala* species, and *Scedosporium apiospermum*. Despite antifungal therapy and optimized pulmonary toilet, he clinically worsened with radiographic evidence of new apical hydropneumothorax, consolidations, and pleural effusions. Consequently, he returned to NIH for further evaluation and management spanning a 13-month hospitalization (Fig. 1).

During that admission, he failed two months of extensive medical management. He was placed on broad spectrum antibiotics and antifungals to cover the microorganisms cultured from sputum (*Serratia* and *Burkholderia)*, bronchoalveolar lavage (*Scedosporium apiospermum, Mycobacterium intracellulaire/chimera*) and pleural effusions (*Scedosporium apiospermum*, *Mycobacterium intracellulaire/chimera)*. He required placement of three chest tubes for drainage of pleural effusions and multiple bronchoscopic placements of endobronchial valves throughout the right lung to seal off air leaks from a right lower lobe pneumatocele complicated by bronchopleural fistula and empyema. He underwent right middle and lower bilobectomy with decortication of right upper lobe and tracheostomy (Fig. 1). Histology of the resected lung tissue revealed granulomatous pneumonitis with numerous necrotizing and non-necrotizing granulomas involving lung and pleura, dense pleural fibrosis and pleural adhesions with granulomas, and small non-necrotizing granulomas within lymph nodes. Immunohistochemical staining revealed extensive lymphoplasmacytic infiltration consisting predominately of CD3^+^ T lymphocytes peribronchially composed of more CD4^+^ than CD8^+^ T lymphocytes in the submucosa and prominent CD20^+^ B lymphocyte nodules around the airways as previously described [12].

**Fig. 3:**
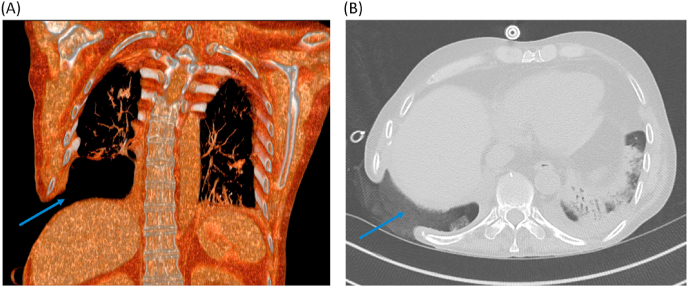
Radiographic appearance of the right chest post-omental flap repair. A. Multi-planar volume reformation (MPVR) image showing a large post-surgical communication between the pleural space and external body surface (blue arrow). B. Axial chest CT illustrating the omental flap, which was harvested from the abdominal cavity and was used to cover the right pleural cavity with a small area remaining open for healing by secondary intention (blue arrow) with packing material noted. Images are from 2015. (For interpretation of the references to colour in this figure legend, the reader is referred to the Web version of this article.)

Post-operatively, he developed persistent right bronchopleural fistulae despite multiple intraoperative patches. After failing a transverse rectus abdominis myocutaneous (TRAM) flap repair, an omental flap closure was ultimately successful (Fig. 3). Secondary to the multiple surgical procedures with extended periods of immobilization, he developed a right parietal embolic stroke -without residual neurological sequelae- and right-sided diaphragmatic dysfunction resulting in persistent hypercapnia responsive to bilevel positive airway pressure (BiPAP).

**Fig. 4:**
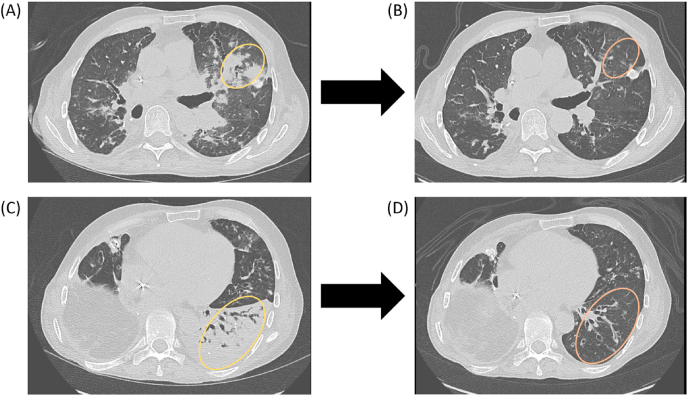
Autoimmune pneumonitis and its improvement following lymphocyte-directed immunomodulation. A-B. Chest CT showing left upper lobe consolidation (A, yellow oval) with interval improvement following immunomodulatory treatment (B, orange oval). C-D. Chest CT showing left lower lobe consolidation with bronchiectatic air bronchograms (C, yellow oval) with interval improvement (D, orange oval) following immunomodulatory treatment. (For interpretation of the references to colour in this figure legend, the reader is referred to the Web version of this article.)

Following surgery, he developed recurrent pulmonary infiltrates and GGOs responsive to steroids. This finding in conjunction with histological evidence of lymphocytic inflammation in the tissue prompted treatment of autoimmune pneumonitis (Fig. 1). He was treated with azathioprine and rituximab and his pneumonitis improved symptomatically, radiographically (Fig. 4), and on functional testing (PFTs improved from FVC 19% and FEV1 20% predicted to 32% and 31% predicted, respectively), and he returned home. Over the next 3 years, he remained clinically and radiographically stable on azathioprine without the need for rituximab redosing, while requiring nightly BiPAP and home oxygen. He remained free from respiratory infections on alternating nebulized tobramycin and amikacin suppression (Fig. 1). He was able to resume regular exercise and returned to work on a part-time basis, resulting in dramatic improvement of his quality of life.

At age 56, he developed progressively worsening dyspnea with mild exertion, increased sputum production, and increasing home oxygen requirements (Fig. 1). Upon his final admission at our institution, a CT scan elucidated new infiltrates and a sputum culture grew MSSA, *Pseudomonas aeruginosa, Aspergillus nidulans, Aspergillus fumigatus, and Stenotrophomonas maltophilia* which were treated with 8 weeks of meropenem, minocycline, and posaconazole. Upon completion of antibiotic therapy and confirmation of radiographic improvement of pulmonary infections, he received rituximab for treatment of underlying autoimmune pneumonitis flare (Fig. 1). Following discharge, he developed a urinary tract infection with *Pseudomonas aeruginosa,* necessitating an extended course of meropenem. While on meropenem, he developed a new consolidation along with worsening hypoxia and dyspnea. Upon admission to an outside hospital, respiratory cultures grew a multi-drug resistant *Pseudomonas aeruginosa* from both sputum and urine. Despite maximal medical therapy, he developed sepsis resulting in multiorgan system failure and death (Fig. 1).

## 3 Discussion

We present this case report of an APECED patient as an example of the severe bronchiectasis-related structural lung disease that can complicate APECED pneumonitis leading to significant morbidity and mortality. Our patient first developed signs and symptoms concerning for pneumonitis early in life before meeting the APECED clinical diagnostic criteria [12]. Consequently, his lung disease was misdiagnosed as asthma and bronchitis for over 40 years, resulting in delayed initiation of immunomodulatory treatment and development of severe bronchiectasis and cavitary lung lesions. This ultimately predisposed him to colonization by resistant microorganisms and recurrent bacterial, fungal, and mycobacterial infections, further complicating medical management and necessitating multiple surgical interventions. Although a histological diagnosis of pneumonitis helped with initiation of lymphocyte-directed immunosuppression which remitted the pulmonary autoimmunity for four years, such therapy did not reverse his structural lung disease or the bronchiectasis-associated infections by resistant microorganisms, which contributed to his death.

This case underscores the critical importance of early diagnosis and treatment of APECED and associated life-threatening pneumonitis to prevent structural lung destruction, recurrent infections, dependence on home oxygen therapy, antibiotic resistance, invasive surgeries and their complications, and death. Early diagnosis relies on recognition of APECED and its associated symptoms, which can be difficult due to its many endocrine and non-endocrine manifestations along with its variability in clinical presentation and severity. To improve recognition and facilitate earlier diagnosis, it has been proposed to expand the diagnostic criteria from the classic triad (two out of the three: CMC, hypoparathyroidism, adrenal insufficiency) to include an adjunct triad of urticarial eruption (APECED rash), intestinal dysfunction, and enamel hypoplasia [11,17]. In a prospective observational study of APECED patients, adoption of these expanded criteria would have accelerated diagnosis by about four years, facilitating earlier treatment and allowing for potential prevention of APECED-associated complications in a large number of patients [11]. Future studies are needed to confirm the validity of these proposed criteria. Importantly, clinicians should maintain a high index of suspicion for APECED when children develop serial autoimmune manifestations, particularly in combination with the classic or adjunct triad manifestations. Earlier diagnosis of APECED could allow for preemptive immunosuppression prior to multiorgan autoimmunity development, including pneumonitis [8].

Pneumonitis is a common manifestation of APECED that presents early in life with chronic cough and radiographic abnormalities and can progress to irreversible bronchiectasis, acting as a nidus for recurrent lung infections [12,17,18]. In a natural history study on APECED, chronic cough was found to be an early, frequently occurring symptom that persisted, in some cases, over 10 years before APECED pneumonitis was eventually diagnosed [12]. Similar to our patient, patients with confirmed APECED are often misdiagnosed with asthma and/or bronchitis resulting in delays of pneumonitis diagnosis and treatment, thereby increasing the risk of developing structural lung disease and its associated morbidity and mortality. A non-contrast chest CT is the most sensitive diagnostic test and identifies all patients with APECED pneumonitis, including those without symptoms and/or patients who do not harbor autoantibodies against BPIFB1 and/or KCNRG [12]. Therefore, it is recommended that all APECED patients undergo periodic screening with chest CT to achieve early diagnosis of APECED pneumonitis [12,17]. Moreover, a high index of suspicion for APECED is required by primary care physicians, pediatricians, and pulmonologists in children and adults who develop chronic respiratory symptoms in the setting of CMC and/or autoimmune manifestations to help avoid delays in diagnosis and treatment.

## 4 Conclusion

• When untreated, APECED pneumonitis can cause progressive irreversible structural lung disease, leading to chronic and recurrent infections, hypoxemic respiratory failure, and death.
• In patients not diagnosed with APECED, the presence of pneumonitis in combination with other autoimmune manifestations should raise suspicion for APECED.
• Because of the potential catastrophic sequelae of untreated lung disease, all patients with confirmed APECED require periodic chest CT imaging to assess for the radiologic findings of previously underrecognized pneumonitis, particularly when respiratory symptoms arise.
• If discovered early, pneumonitis could be successfully treated with lymphocyte-targeted immunomodulation, preventing the cycle of inflammation and infection that leads to irreversible lung tissue destruction.

## Author contributions

SAK, HMD, and EMNF reviewed and evaluated all records and wrote the case report; DSS, KNO, JGW, MSL, and LRF provided clinical care and critically reviewed the manuscript. All authors approved the final manuscript as submitted and agreed to be accountable for all aspects of the work.

## Funding

This work was funded, in part, by the Division of Intramural Research, 10.13039/100000060NIAID, 10.13039/100000054NCI, 10.13039/100000050NHLBI, and 10.13039/100000098NIH Clinical Center, NIH.

## Declaration of competing interest

Dr. Les Folio is a primary investigator in a corporate research agreement with Philips Health (Amsterdam, Netherlands), the PACS used in this initiative. The other authors declare that the research was conducted in the absence of any commercial or financial relationships that could be construed as a potential conflict of interest.
